# Temperature and Emergency Department Visits: Present-Day Associations and Future Projections

**DOI:** 10.21203/rs.3.rs-7584734/v1

**Published:** 2025-10-09

**Authors:** Chad Milando, Ian Sue Wing, Gregory A. Wellenius

**Affiliations:** Boston University School of Public Health; Boston University; Boston University School of Public Health

**Keywords:** Temperature, Emergency Department Visits, Projected Future Impacts, Distributed Lag Nonlinear Models, Case-crossover

## Abstract

High ambient temperature (i.e., “heat”) is associated with increased rates of death. The association between heat and illnesses that result in emergency department (ED) visits is less well characterized: most studies have examined heat impacts in the summer months, for a subset of health outcomes, or at limited spatial scales. This work characterizes the relationship between temperature and all-cause ED visits across the entire year and at large spatial scale, and projects ED visits associated with future temperature change. We analyzed health data from 21,090,141 commercially insured individuals across 2,161 counties in the contiguous US from 2010 to 2021. We used distributed lag non-linear models (DLNM) to fit the exposure-response relationship in the historical period (2010–2021), then used projected mid-century (2040–2050) temperatures from global climate models across three Shared Socio-Economic Pathways to project future all-cause ED visits. Increasing daily maximum temperature were associated with increased ED visits throughout the year and across the US. ED visits increased 1.3% per year under the highest emission scenario and more than half of the counties in the study experienced 27 or more days per year of high healthcare utilization under future climate scenarios, an increase of 50% over present day conditions. These findings clarify the exposure-response relationship between daily maximum temperature and ED visits as distinct from the relationship between temperature and mortality. They also emphasize that future healthcare systems will need to accommodate more baseline ED visits throughout the year and a higher frequency of days with extremely high ED visit rates.

## INTRODUCTION

High ambient temperature (i.e., “heat”) is associated with excess death.^1–7^ In the United States (US) from 1997 to 2006, extreme heat was associated with approximately 10,000 deaths annually.^8^ The 2022 European heatwave was associated with approximately 61,000 excess deaths.^9^ The association between heat and illness that results in emergency department (ED) visits or hospitalizations is less well-known, but no less important; such events represent major life disruptions and utilize substantial healthcare resources.^10^ In studies of heat and illness limited to specific populations in summer-time and at the regional or sub-regional scale, heat has been associated with increased ED and hospitalization rates.^11–16^ However, a comprehensive analyses has yet to be performed that includes a population of all ages across a large spatial area, and for all-cause outcomes occurring across all months of many years.

Enhancing understanding of the associations between ambient temperature and health impacts is critical for healthcare management in a warming climate.^17,18^ The association between extreme temperature and mortality is “U-shaped”: days of extreme heat and cold are associated with excess deaths compared to days with moderate temperatures.^19–22^ In the US, climate models project fewer extremely cold days and more extremely hot days in the coming decades.^23,24^ Therefore, a reduction in the number of extremely cold days is estimated to be associated with a reduction in cold-related deaths, and an increase in heat-related deaths, thus attenuating the overall impact of warmer temperatures on mortality.^19–22^ The same pattern does not necessarily hold for illness. Results are mixed as to whether the association between year-round temperature and illness is “U-shaped” or monotonically increasing.^21,25–30^ In the latter case, warmer temperatures in any season could be associated with more frequent illnesses, resulting in greater healthcare utilization and costs.

To address this evidence gap, we assessed the relationship between daily maximum temperatures and ED visits among more than 21 million enrollees in commercial or Medicare Advantage health insurance plans across the contiguous US from 2010 to 2021. We then projected ED visits under three scenarios of warming at mid-century (2040–2050). Our findings clarify the exposure-response relationship between year-round temperature and ED visits across the entire contiguous US and provide important projections of the scale of increased ED visits due to the near-certain increase in temperatures in the future.

## METHODS

### Outcomes

**Figure 1:**
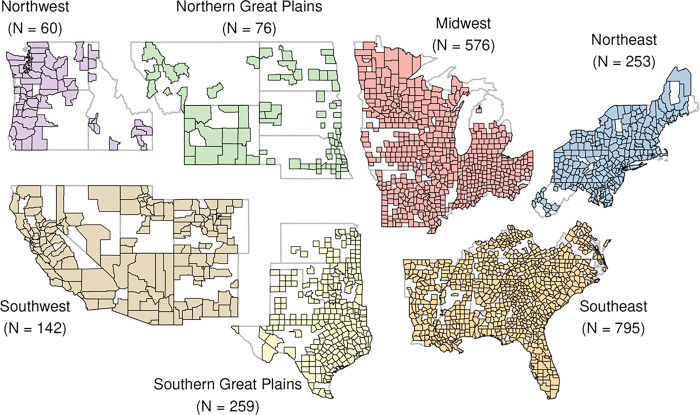
US counties with more than 500 annual average Optum Labs Data Warehouse (OLDW) enrollees. This study included 21,090,141 OLDW enrollees in 2161 US counties from 2010 to 2021. Regions defined according to the 4^th^ National Climate Assessment (NCA4): Southeast, Southwest, Northern Great Plains, Southern Great Plains, Midwest, Northeast, and Northwest.

We studied all-cause ED visits among people of all ages across the contiguous US enrolled in commercial and Medicare Advantage health insurance in the Optum Labs Data Warehouse (OLDW)^®^ from 2010 to 2021, a total of 21,090,141 enrollees across 2,161 US counties (Fig. 1). We limited our study domain to US counties with more than 500 enrollees per year and adjusted ED visit counts within each year by annual enrollee levels. We then aggregated counts by county and region of residence, with regions defined according to the 4th National Climate Assessment (NCA4): Southeast, Southwest, Northern Great Plains, Southern Great Plains, Midwest, Northeast, and Northwest.^31^ This use of these pre-existing, de-identified data was approved by the Institutional Review Board of Boston University (IRB number: H-40274), and informed consent was waived. All methods were carried out in accordance with relevant guidelines and regulations.

### Exposures

Present-day daily maximum temperatures were estimated from the Copernicus Climate Change Service, a dataset known as ERA5^32^ that has been population-weighted to the US county scale^33^ and utilized in studies temperature and health.^34^

For projections of future daily maximum temperatures, we gathered results from 32 global climate models (GCMs) across 3 different Shared Socio-Economic Pathways (SSPs) for projected mid-century (2040–2050) future temperatures (**Table S1**).^35^ The SSP scenarios are characterized by the relative level of global warming: SSP5 (highest warming), SSP3 (moderate to high warming), and SSP2 (moderate warming).

### Exposure and outcome association

**Figure 2:**
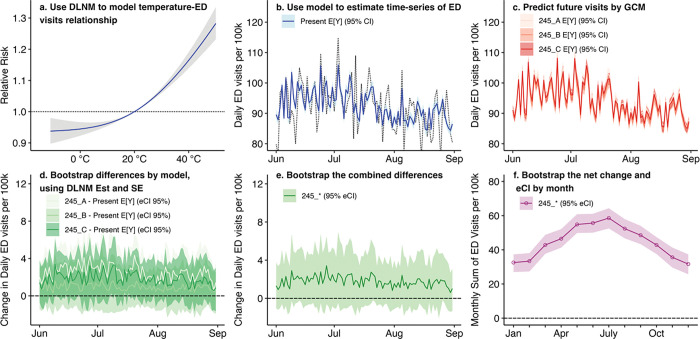
Graphical representation of the statistical methods used in this paper (using dummy data representing a single county). Panel (a) shows the exposure response curve resulting from a distributed lag non-linear model (DLNM). Panel (b) displays how this model is applied to the current dataset to create a multi-year timeseries of expected values of ED visit counts in the present day data (Present E[Y]). For panel (b) and panels (c), (d) and (e), only the summer months of a single year of the multi-year time-series is shown. Panel (c) shows how the temperature change from different global climate models (GCMs) within a Shared Socio-economic Pathway (SSP) is used to estimate the expected value of future ED visits resulting from a time-series of present-day temperatures shifted by the estimates of each GCM (e.g., 2–4.5_A, 2–4.5_B, and 2–4.5_C all represent different GCMs under SSP 2–4.5). Panel (d) shows the bootstrapped difference between present and future expected values and the empirical confidence intervals (eCI 95%). Panel (e) shows the daily bootstrapped difference across all GCMs within an SSP (i.e., the differences in 2–4.5_A, 2–4.5_B, and 2–4.5_C are combined into 2–4.5_*). Panel (f) shows the mean and empirical confidence interval (eCI) of the combined difference at the average monthly level across the entire timeseries, obtained by bootstrapping and summing values by region and month.

Estimating the exposure-response relationship and projecting future impacts used a sequence of modeling methods, outlined in Fig. 2. We first fit distributed lag non-linear models (DLNM) to estimate the relationship between daily maximum temperature and ED visits (Fig. 2. **Panel a**). We used a single-stage distributed lag non-linear modeling framework with a quasi-Poisson model; this approach has been extensively used in environmental epidemiology related to temperature exposure.^36^ For each county, we created a basis matrix for the temperature vector using natural cubic splines with internal knots at the 10th, 75th, and 90th percentile of daily county temperature in the present-day period. We extended the boundary knots to include the projected future temperature extremes. We then created a basis matrix for the lag structure, assuming again a natural cubic spline, with a maximum lag of 5 days (and sensitivity tests for 3 and 10 days), and three internal knots equally spaced on the time axis on the log scale. We then created a crossbasis for lagged temperature from these individual bases, using the dlnm package.^37^ Novel to this work was adapting the method of how the future temperature basis was created. Instead of creating a new basis directly from the vector of future temperatures,^38^ we instead rearranged rows of the present-day exposure matrix depending on the county-specific temperature percentile each day fell in (using bins of 1°C). By doing so, we ensured that the regression model coefficients fit using present-day data could be applied to a future temperature time-series while maintaining the present-day exposure-response relationship. To derive the exposure-outcome relationship, we fit a quasi-Poisson model of county-specific all-cause ED visits using the present-day lagged exposure matrix, a dummy variable for day of week and a natural cubic spline to account of varying time trends (with 8 degrees of freedom per year). All analyses were performed in R version 4.2.1.^39^

### Projection of future outcomes

We then used the coefficients from the present-day model and the future temperature exposure matrix to create a future time-series of the expected value of daily ED visits with standard errors for each modeled point (Fig. 2. **Panel b**). To view the exposure-response relationships by NCA4 region, we reduced the model output along the lag dimension and calculated the relationship within regions using the mixmeta package.^36^

Using the change in daily maximum temperature by GCM and SSP, we then created new multi-year timeseries of the expected ED visits among the present-day population but with the projected mid-century temperatures (Fig. 2. **Panel c**.). Each of these future time-series also had a point estimate and standard error.

For each GCM within each SSP, we then calculated the difference in present day versus future ED visits using a bootstrap process. We used the present day and future expected ED visit counts and standard errors as parameters for two normal distributions and created 250 estimates of the difference between present day conditions and each GCM output (Fig. 2. **Panel d**.). We then calculated a combined difference and empirical confidence interval for each county and GCMs within each SSP by performing another bootstrapping process (Fig. 2. **Panel e**.). Thus, for each combination of county, GCM, and SSP, we had a multi-year time-series (and empirical confidence interval) for the daily change in ED visits that incorporated uncertainty from both the DLNM models and median daily estimates of GCMs. Resulting confidence intervals were narrow, reflecting the distribution around the median estimate, rather than the range across GCMs. In estimating uncertainties, we followed approaches similar to the temperature and mortality literature.^20^

We created summary estimates of the sum of monthly changes in ED visits within NCA4 regions (Fig. 2. **Panel f**.). We sampled daily estimates of the difference between present and future ED visit within each combination of month, county, and SSP, then summed to the region and SSP level. Thus, for month, by season, or for a full year, we created estimates of the new ED visits that would occur under mid-century temperatures, all else being equal. To calculate the estimated change in ED visits, we compared averages of total ED visits per year.

Finally, we estimated how much more often surges in healthcare utilization would occur under future climate scenarios. Specifically, we estimated the number of additional days on which the number of ED visits exceeded the county and year-specific 95th percentile of the distribution of daily ED visit counts By definition, during the present-day period (2010 through 2021) there are 18 high healthcare utilization days that exceed the threshold defined by the 95th percentile of daily ED visits per county and year. These ‘high utilization’ days help estimate the increased burden on the healthcare system due to an increased frequency of extremely hot temperatures.

## RESULTS

**Figure 3:**
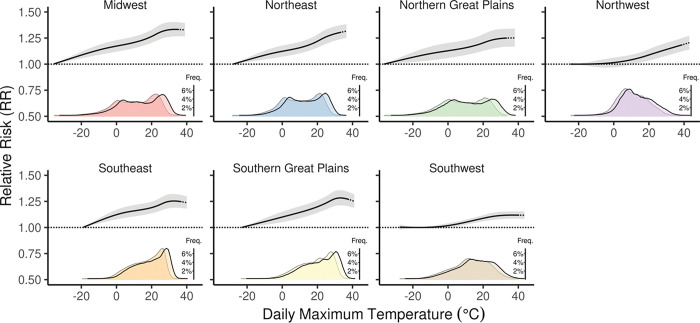
The exposure-response relationship and 95% confidence interval (CI) for daily maximum temperature and the relative risk of all-cause Emergency Department (ED) visits for OLDW enrollees in 2161 counties in the contiguous US from 2010 to 2021. The reference point for estimating the relative risk in each NCA4 region is represented by the temperature with the lowest relative risk of an ED visit. The dotted portion of each exposure-response curve shows the linear projection for temperatures above the range of temperatures in 2010 to 2021. The density plots beneath show the current (bordered in grey) and future (bordered in black) temperature distributions given the median of SSP5–8.5 scenarios.

Across the US, increasing daily maximum temperatures was largely monotonically associated with increased relative risk of ED visits, i.e., not “U-shaped” (Fig. 3). Only the Southern Great Plains and Southeast had exposure-response relationships which were elevated for high present-day temperatures but decreased slightly for projected future temperature extremes. We observed similar results at various exposure lag times typically used when examining changes in rates of ED visits due to extreme temperature (3 to 10 days, **Figure S1, S2**).

The highest warming scenario resulted in projected increases in median temperature from 1.6 to 3.3°C in summer and from 1.2 to 2.0°C in winter (**Table S1**). These increases resulted in a 1.3% increase in annual ED visits relative to the present day across the US (Table 1), a which figure translates to roughly 86,000 additional ED visits annually across the US among the commercially insured study population. The largest percentage increases were in regions that experience relatively cool climates today: the Northeast (+ 1.6%), Midwest (+ 1.4%), and Northwest (+ 1.5%). An analogous pattern of results was observed under lower-warming scenarios (**Table S2**). The severity of increases in ED visits varied by month and region (**Figure S3).** In the Midwest, Northwest, and Northeast, the largest projected impacts of warming temperatures on ED visits occurred in the summer months (June through August). Conversely, in the Northern and Southern Great Plains and the Southwest, the largest projected increases in total ED visits occurred in the spring (March through May) and fall (September through November). In the Southeast, the impact of projected warming was relatively constant throughout the year.

**Figure 4:**
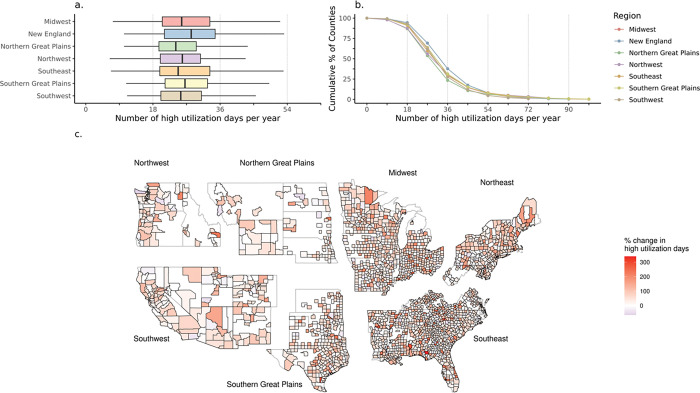
For the high emission scenario (SSP5–8.5), (a) boxplots showing the distribution of future high utilization days per year by NCA4 Region, (b) the cumulative proportion (%) of counties experiencing high utilization days using projected temperatures, and the (c) spatial distribution of county-specific changes (%) in the number of high utilization days. In each plot, a high utilization day is one with an emergency department (ED) visit count greater than the annual 95^th^ percentile of daily ED visits.

Under future projected scenarios, in each year there were many more days of high healthcare utilization that exceeded present-day thresholds (Fig. 4). Under the highest warming scenario, 54% to 69% of all study counties exhibited a greater than 50% increase in high utilization days (more than 27 days), 23% to 38% saw the number of days over their current threshold double (more than 36 days), and 11% to 17% of counties experienced 45 or more high utilization days (a 150% increase). The Northeast region had the highest increase in high utilization days, while the Northern Great Plains had the smallest increase. This pattern of results was similar but of a lesser magnitude for the lower-warming scenarios (**Table S3**).

## DISCUSSION

Among more than 21 million people with commercial health insurance across the lower 48 US states, we observed a positive and monotonic association between daily all-cause ED visit rates and daily temperatures, not “U-shaped” as for heat and mortality. We subsequently projected that increases in temperature in midcentury resulted in substantial increases in annual ED visits, 1.3% in the highest warming scenario. For context, this increase is similar in magnitude to the overall increase in the US ED visit rate from 1997 to 2007 (a 1.2% increase, from 352.8 to 390.5 visits per 1,000 persons per year).^40^ Finally between half and two-thirds of counties were projected to experience at least a 50% increase in high ED utilization days by midcentury, with impacts concentrated in cooler northern regions.

The present paper adds to a small number of studies on the exposure-response relationship between heat and non-fatal health impacts. Most similar to ours is an investigation of current and future ED visits for hyperthermia in 136 US urban areas.^41^ That study found largely monotonic increases in May-September hyperthermia ED visit rates for adults 18 to 64 that translated to a projected increase in ED visit rates for hyperthermia. Hess et. al., (2014) used state-level ED records to show a linearly increasing relationship between average annual temperature anomaly and annual ED visits for heat-related illness.^42^ Finer geographic scale studies have also demonstrated monotonic or “J-shaped” associations between increased temperature (with lags of 3 to 7 days) and non-fatal health impacts: in Shanghai and Pudong New Area, China^25,26^ and in the US in California, Rhode Island, Massachusetts, and North Carolina^27,28,43,44^ Different from our result was a study across 12 cities in China that used a lag period of 32 days and showed a “U-shaped” exposure-response relationship between heat and ED visits.^30^ The appropriate length of the lag time for calculating non-fatal health impacts of heat (as compared to fatal impacts) is an open question, especially given the potential for future heatwaves with multiple consecutive extreme heat days.^45,46^

We can also situate our results in the context of heat-and-health research for mortality and for ED visit surges. Our findings with ED visits are similar to research on heat and mortality that show higher mortality impacts in high-latitude locations where infrastructure to adapt to extremely hot days may be lacking.^2^ The impact on ED visit surges due to extreme heat (> 35°C) was also quantified in Brisbane, Australia; by 2030, days of extreme heat were projected to increase ED visit usage by up 200% above present-day levels. ^47,48^

To enable this work, we modified an existing method for projecting the future morbidity consequences of increased temperature.^38^ The commonly-used approach estimates the increase in the *fraction* of observed cases that *would have been attributed to temperature* had temperatures been warmer.^1–7^ By contrast, our interest is estimating the *additional* ED visits that would occur under warmer temperatures, a value which encompasses not only the increased fraction of observed cases but also new cases that did not occur in the historical record. The key modification to the existing method was extending the boundary knots of the present-day temperature basis to include the range of future projected temperatures, and then constructing matrices of lagged temperature exposure for the present-day and future periods from this extended basis – an approach adapted from similar economic literature.^27^

The benefits of our modified approach are two-fold. First, our exposure-response functions for future exposures now include both non-linearities and lagged effects. Although studies of temperature and mortality have shown that the strongest associations between heat and health occur on the day of exposure,^49^ in warmer future climates where contemporaneous and lagged temperatures can exceed historical percentiles, lagged exposures could potentially exert larger adverse health impacts. Second, the method of calculating additional health impacts of future temperatures does not depend on the shape of the exposure response curve. Temperature-mortality studies have exploited the fact that the “U-shaped” exposure-response relationship’s nadir presents a natural point of comparison for the relative risks of colder versus hotter temperatures.^1–7^ However, the monotonicity of our estimated exposure-response curves render estimates of attributable number and attributable fraction using that approach highly sensitive to comparison point selection.^38,43^

Our results are subject to several caveats, all of which constitute opportunities for further inquiry. We did not account for future population dynamics or adaptation.^50^ Although such projections are available,^51^ their use is predicated on our currently limited understanding of how responses differ among demographic subgroups and intersect with susceptible individuals’ capacity to adapt. Our omission of these influences results in relatively narrow confidence intervals for estimated ED visits. A further uncertainty is the extent to which our estimated responses, which reflect the subset of currently-insured individuals, and therefore could underestimate the population incidence of heat-related illness,^52^ are representative of the true response in the broader US population—especially as the latter undergoes heterogeneous region-specific compositional shifts out to mid-century. Our modeling method assumes that the underlying causes of ED visits scale with temperature. Established practice treats the uncertainty associated with this assumption as being captured by our standard errors, which are small, and temperature differences across warming scenarios, which are larger.^38^ We note however that there is an additional source of uncertainty, in the form of the dispersion of simulated temperatures and associated ED visits across GCMs within scenarios.^53^ For the sake of concision, we leave characterization of that uncertainty to follow-on work. Our case-crossover model also did not incorporate specific adjustments for the COVID-19 pandemic. However, we note that any attendant biases would affect both present and future projections, and thus exert little influence on the changes we estimate here. Computational constraints also precluded meta-analysis of county-specific modeled coefficients that were *not* cross-reduced, limiting our ability to use best linear unbiased predictions of county-specific exposure response functions.^36^

Our findings clarify the exposure-response relationship between year-round temperature and ED visits across the entire US and provide estimates for which regions may be at highest risk of increased ED visits due to increased temperatures in the future, absent other adaptation or population dynamics. Of paramount importance is uncovering the origins of monotonicity in the exposure response. It remains to be established whether this phenomenon reflects behavioral influences in the tails of the temperature distribution, or whether different diagnoses or risk factors might be more commonly associated with elevated risk of ED visits on hot days. With temperature increase all but certain,^54^ these findings quantify the need for future healthcare systems to accommodate both more baseline ED visits due to increased heat as well as a higher frequency of days with extremely high rates of ED visits.

## Supplementary Material

Supplementary Files

This is a list of supplementary files associated with this preprint. Click to download.

SRSupp.docx

## Figures and Tables

**Table 1:** Modeled median changes in annual Optum Labs Data Warehouse (OLDW) Emergency Department (ED) visits by NCA4 region due only to Mid-century (2040–2050) temperatures from Shared Socio-economic Pathway (SSP) model 5–8.5. Present-day and future modeled ED visits are estimated using distributed lag non-linear models (DLNMs) and using 2010–2021 ED visits among 21,090,141 OLDW enrollees in 2,161 US counties. The change in annual average ED visits was estimated using bootstrap methods.

NCA4 region	N counties	Annual Avg. OLDW Population (2010–2021)	Annual avg. ED visits (2010–2021)	Annual avg. ED visits *modeled* (2010–2021)	Change in Annual avg. ED visits *modeled* (2040–2050)	*Average modeled* % change in annual ED visits
Midwest	576	5,163,809	1,718,395	1,718,392 (1,718,247, 1,718,549)	+ 24,772 (24,497, 25,055)	+ 1.4
Northeast	253	3,483,720	1,055,423	1,055,422 (1,055,312, 1,055,526)	+ 16,712 (16,493, 16,914)	+ 1.6
Northern Great Plains	76	286,049	83,857	83,855 (83,825, 83,887)	+ 1,022 (964, 1,083)	+ 1.2
Northwest	60	501,741	130,086	130,084 (130,046, 130,126)	+ 2,005 (1,935, 2,083)	+ 1.5
Southeast	795	5,778,368	2,186,146	2,186,152 (2,186,014, 2,186,320)	+ 23,732 (23283, 24168)	+ 1.1
Southern Great Plains	259	2,937,845	907,101	907,092 (906,978, 907,228)	+ 10,873 (10,654, 11,118)	+ 1.2
Southwest	142	2,938,609	691,344	691337 (691,245, 691,433)	7,201 (7,009, 7,374)	+ 1.0
All	2,161	21,090,141	6,772,351	6,772,338 (6,772,077, 6,772,616)	86,298 (85,688, 87,007)	+ 1.3

## Data Availability

The data that support the findings of this study are available from Optum Labs but restrictions apply to the availability of these data, which were used under license for the current study, and so are not publicly available. Due to the terms of the contractual data use agreement, the authors are not able to release data to others. Interested parties may contact Optum Labs directly to explore licensing access to the data. The code to recreate Figure 1 and demonstrate our approach are available on Github: www.github.com/cmilando/FuturePredMethods.
